# Topology based identification and comprehensive classification of four-transmembrane helix containing proteins (4TMs) in the human genome

**DOI:** 10.1186/s12864-016-2592-7

**Published:** 2016-03-31

**Authors:** Misty M. Attwood, Arunkumar Krishnan, Valentina Pivotti, Samira Yazdi, Markus Sällman Almén, Helgi B. Schiöth

**Affiliations:** Department of Neuroscience, Functional Pharmacology, Uppsala University, BMC, Box 593, 751 24 Uppsala, Sweden; Institutionen för neurovetenskap, BMC, Box 593, 751 24 Uppsala, Sweden

**Keywords:** Human proteome, Four transmembrane, 4TM, Function, Topology prediction, Structure function, Cancer, Drug targets

## Abstract

**Background:**

Membrane proteins are key components in a large spectrum of diverse functions and thus account for the major proportion of the drug-targeted portion of the genome. From a structural perspective, the α-helical transmembrane proteins can be categorized into major groups based on the number of transmembrane helices and these groups are often associated with specific functions. When compared to the well-characterized seven-transmembrane containing proteins (7TM), other TM groups are less explored and in particular the 4TM group. In this study, we identify the complete 4TM complement from the latest release of the human genome and assess the 4TM structure group as a whole. We functionally characterize this dataset and evaluate the resulting groups and ubiquitous functions, and furthermore describe disease and drug target involvement.

**Results:**

We classified 373 proteins, which represents ~7 % of the human membrane proteome, and includes 69 more proteins than our previous estimate. We have characterized the 4TM dataset based on functional, structural, and/or evolutionary similarities. Proteins that are involved in transport activity constitute 37 % of the dataset, 23 % are receptor-related, and 13 % have enzymatic functions. Intriguingly, proteins involved in transport are more than double the 15 % of transporters in the entire human membrane proteome, which might suggest that the 4TM topological architecture is more favored for transporting molecules over other functions. Moreover, we found an interesting exception to the ubiquitous intracellular N- and C-termini localization that is found throughout the entire membrane proteome and 4TM dataset in the neurotransmitter gated ion channel families. Overall, we estimate that 58 % of the dataset has a known association to disease conditions with 19 % of the genes possibly involved in different types of cancer.

**Conclusions:**

We provide here the most robust and updated classification of the 4TM complement of the human genome as a platform to further understand the characteristics of 4TM functions and to explore pharmacological opportunities.

**Electronic supplementary material:**

The online version of this article (doi:10.1186/s12864-016-2592-7) contains supplementary material, which is available to authorized users.

## Background

Membrane proteins are essential in several cell processes and participate in a wide variety of functions, including playing pivotal roles in signaling pathways, acting as regulatory elements, functioning as receptors, and also facilitating the transport of ions and molecules across the impermeable lipid bilayer [1]. Due to their diverse and important functional activities, membrane proteins serve as major targets for pharmaceutical industries [2, 3]. Approximately 20–30 % of most animal proteomes are transmembrane proteins, which in humans amounts to ~5500 proteins [4–6]. The preponderance of membrane proteins attach to the membrane with transmembrane α-helices, while the rest are characterized by transmembrane β-strands forming β-barrels. Previous topology based analyses of membrane proteins in the human genome have shown that the largest category of α-helical membrane proteins is composed of only one transmembrane spanning helix, while the second largest category includes the two transmembrane helix containing proteins [5] [7]. In particular, several studies point toward a strong correlation between membrane protein structure and function and that the number of transmembrane helices substantially determines what function the protein carries out [5, 8–10]. Moreover, analyses of structural topologies of membrane proteomes have shown that the C-terminus is predominantly located in the inside of the membrane, as found across humans [5], *Escherichia coli* [11] and *Saccharomyces cerevisiae* [12], and can be engaged in activities such as stabilization, signaling, protein interactions, and channel gating amongst others [13, 14]. This C-terminus localization is ubiquitous as the majority of proteins containing odd numbers of helices, such as those in the large 1TM and 7TM groups, have intracellular C-termini with N-termini in the extracellular environment, and protein groups with even helical numbers have a greater amount of both termini located intracellularly. Our earlier complete curation of the membrane proteome in the human genome showed that certain membrane topologies are more common for specific functions such as enzymatic activity, receptors, and transporters [5]. For example, the majority of receptors fall into two major categories: those that contain either one transmembrane helix (TM), or the well-studied 7TM G protein-coupled receptor (GPCR) group [15–17] which composes 67 % of human receptors [5]. This means that receptors have a greater number of their N-termini in the extracellular environment, which functions in activities such as protein interactions, protein targeting, and signaling [18]. Transporters, another well-researched group, tend to have six or more membrane-spanning helices, such as the solute carriers (SLC) that contain 10–14TM [19]. However, membrane bound enzymes generally contain fewer helices and they largely fall within 1TM or 2TM containing proteins.

Among these TM families that are categorized by the number of α-helices that span the membrane, the four-transmembrane helix containing proteins are characterized by an array of protein families, such as the neurotransmitter gated ion channels, claudins, connexins, and tetraspanins, that display large degrees of diversity in their functions. Neurotransmitter gated ion channels (NGIC) mediate a rapid transmission of signals at chemical synapses and are a heavily investigated group that act as both receptors and transporters [20]. They have four evolutionarily and structurally related 4TM families that include 45 members [21] plus one more family consisting of one gene, zinc activated ligand-gated ion channel (*ZACN*) [22]. Claudins are another major 4TM family and are one of the main structural components of tight junctions and mediate cell-cell adhesion. There are currently 27 mammalian claudin members that are found solely in epithelial cells. [23]. They contain the PMP22_Claudin domain (PF00822) which is a member of the Transporter superfamily clan, as well as two conserved cysteine residues in extracellular loop one, and most claudins contain a PDZ binding motif at the C-terminus position [24]. Connexins, which are also members of the Transporter superfamily clan, have been extensively studied as they are the structural components of gap junctions, which are involved in cell-cell communication. There are 21 different connexin proteins described by several conserved features: a four membrane-spanning domain (PF00029) and a conserved three-cysteine residue domain (PF10582) found on each of the two extracellular loops. And finally, the Tetraspanins include 33 members in which some members have a wide and abundant distribution throughout tissues and others are more selectively expressed [25]. These ‘true’ tetraspanins (to differentiate from proteins with 4TMs that can also be called tetraspanins) contain the tetraspanin functional domain (PF00335), which is a member of the Tetraspanin-like clan, as well as a conserved CCG motif with 4–6 conserved extracellular cysteine residues [26].

Within these larger protein families, selected proteins have been well characterized, however there still remain many 4TM proteins that have yet to be fully classified. To the best of our knowledge, the complete complement of four-transmembrane helical containing proteins has not been characterized before in any vertebrate genome, and curation of the 4TM component of the human genome can provide an important basis to understand the diversity of the important 4TM proteome. First, we aim to provide a qualitative functional classification of the complete 4TM dataset screened from the latest release of the human genome (GRCh38.p3). Second, we sought to analyze whether there is any correlation between the topological orientations (N-terminus inside or outside of the membrane) and their functional activities, that is if certain topologies are favored for certain functional classes. Third, we aim to identify how many of these 4TM proteins are associated with diseases and how many of them are potential drug targets.

Here, we present the first complete gene repertoire and detailed functional classification of the human four membrane-spanning protein dataset.

## Results

### Identification and curation of the 4TM dataset

**Fig. 1:**
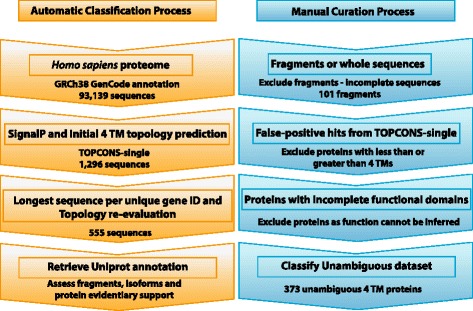
(Parts A and B). Automatic and manual classification process. Part 1A: Schematic diagram of the automatic classification process. The first step in the automatic characterization process included downloading the *Homo sapiens* proteome from the Genome Reference Consortium human genome 38 (GRCh38) release with the GenCode annotation information. SignalP standalone was used to assess and excise any signal peptides. The four membrane-spanning regions were predicted using TOPCONS-single, which comprises five different prediction tools and returns a consensus decision of the number of transmembrane areas. The longest sequence for each genomic location, or gene, was then selected and then those sequences were re-evaluated with TOPCONS-single. Uniprot, which is a large comprehensive repository of protein sequences with both manually curated and automatically generated annotations, was used to download associated annotations for each sequence in the predicted dataset. Part 1B: Schematic overview of the manual curation process for each individual sequence. The purpose of the individual sequence inspections was to ensure the 4TM dataset was composed of valid proteins with four membrane-spanning regions so that the function could be inferred and described. The predicted dataset was evaluated for validity and reliability using the CCDS dataset. The predicted dataset includes proteins that may be fragments, possible false-positive hits from TOPCONS-single, and protein isoforms that contain incomplete functional domains. The Uniprot annotation data included information such as whether the sequence was a fragment or not, any associated Pfam functional domains or families, the Transportation Classification (TC) number, the Enzyme Commission (EC) number, as well as Gene Ontology annotation terms

A combination of *in silico* automatic classification followed by individual sequence curation was used to analyze proteins with four membrane-spanning regions (see Fig. 1: Parts A and B, and Methods section for details). The initial human proteome contained 93,129 protein coding sequences, and after excising signal peptides 1,296 4TM sequences were initially predicted using TOPCONS-single. The predicted dataset was reduced by selecting the longest sequence length per unique gene (using ENSEMBL gene identification), re-evaluated by TOPCONS-single, and the non-redundant dataset includes 555 proteins. This dataset does include proteins that may have incomplete sequences that do not contain initial and/or terminal residues; that are possibly false-positive hits from TOPCONS-single; as well as proteins that are isoforms and contain incomplete functional domains. Additionally, the proteins need to be evaluated for annotation quality and protein validity, i.e. that they have recognized protein-coding descriptions with acceptable transcriptional support.

The predicted proteins were evaluated for validity and reliability through comparison with the CCDS dataset and then further manually refined (Fig. 1B). The 4TM dataset includes 101 sequences identified as fragments. There are 106 proteins established as isoforms that include 88 proteins that contain Pfam functional domains that are cited through literature as containing more than four helices, indicating the protein has an incomplete domain and consequently functionality cannot be inferred. Additionally, 15 proteins have been identified through literature and database resources as suspected false-positive hits from TOPCONS-single. None of these proteins are included in the 4TM unambiguous dataset. Forty additional proteins were manually evaluated and added to the dataset to include members of known 4TM protein families (for example, claudins and tetraspanins). The final dataset includes 373 unambiguous 4TM proteins, representing a single protein product from each gene. These 373 proteins are categorized into five major groups based on functional, structural, and/or evolutionary similarities: Transporters (66), Enzymes (45), Dual Function (47), Receptors (2), and Miscellaneous (213). The proteins identified as Miscellaneous are further classified into subgroups with similar functions based on GO terms, Pfam domain descriptions, and protein family descriptions.

### Functional classifications

#### Transporters

**Fig. 2:**
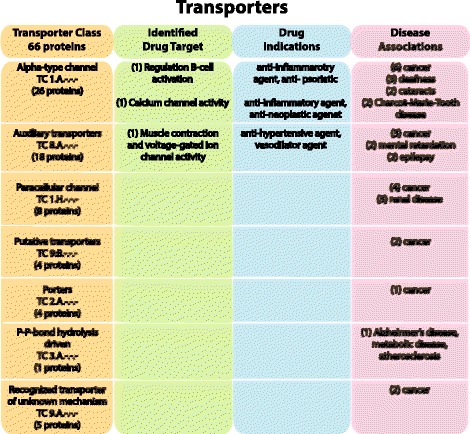
The human 4TM Transporter class. The figure shows the 66 proteins of the Transporter class that are further categorized into seven different subclasses. The Transporter Classification number (TC) is determined by the Transporter Classification Database, which has been cross-referenced through Uniprot to obtain the TC number associated with the protein. The TC system is specific for membrane transport proteins and includes both functional and phylogenetic information in the numbering information. The transporter proteins identified as drug targets and the drug indications are also presented. An updated dataset of all current targeted and potential proteins and genes involved in drug studies or experimentation was used to identify proteins that are drug targets as well as the drug indications. In addition, the top common gene-disease associations are displayed for each subgroup, with the number of proteins involved in parenthesis. Three different resources were used to identify gene-disease associations: the Online Mendelian Inheritance in Man (OMIM) database; the Functional Disease Ontology (FunDO) resource; and the Jensen Lab Diseases database (see Methods for details). Proteins that participate in transport activity but do not have an associated TC number are not included in this class, but rather in a subgroup of the Miscellaneous proteins. The number in parenthesis represents the number of proteins that have been identified in that group

The Transporter category is the largest functional group with 66 proteins that are further divided into seven different classes (Fig. 2). Proteins identified with a Transport Classification number (TC) are included in this functional class. The *alpha-type channel* (TCDB ID: 1.A.-.-.) is the largest group and contains 26 proteins. *Alpha-type channels* transport solutes such as potassium, calcium, sodium, and chloride ions through transmembrane pores or channels via an energy-independent process [27]. Eight of these proteins function as subunits in gap junctions, where six are connexins and two are pannexins. There are two proteins within this group that are identified as drug targets: B-lymphocyte antigen CD20 [SwissProt: P11836] is involved in the regulation of B-cell activation and proliferation; and Calcium release-activated calcium channel protein 1 [SwissProt: Q96D31] which mediates calcium influx following depletion of calcium stores [28]. Sixteen genes in the alpha-type channel proteins are identified in various disease conditions and several of the most common disorders are displayed in Fig. 2. For a complete overview of the dataset including gene-disease associations, see Additional file 1.

Eighteen proteins are identified as *auxiliary transport proteins* (8.A.-.-.-) that facilitate transport across membranes but do not directly participate in the transport process [27]. Eleven of these proteins belong to the Transporter superfamily clan and six proteins are true tetraspanins. All eight proteins that belong to the *paracellular channel: claudin tight junction* class (1.H.-.-.-) contain the PMP22_Claudin domain (Pfam family: PF00822) and are members of the previously described claudin proteins. Paracellular transport transpires outside of cells and solutes passively follow concentration gradients or transcellular electrical potentials [27]. Four proteins are identified as *putative transport proteins* (9.B.-.-.-) where transport function has been suggested for them, but evidence is not yet complete and these proteins will eventually be classified elsewhere or eliminated [27]. Two of these putative transporters contain the MARVEL domain (PF01284): Occludin [SwissProt: Q16625] is a member of the tight junction associated MARVEL proteins (TAMP) [29] and is involved in several diseases including cancer and neurologic disorders; and Synaptophysin [SwissProt: P08247] is involved with structural functions and in targeting vesicles to the plasma membrane [30] and is also involved in cancer, Alzheimer’s disease, schizophrenia, and mental retardation. Four proteins are *porters* (2.A.-.-.-) that use a carrier-mediated process to catalyze solutes through the membrane [27]. *P-P-bond hydrolysis-driven transporters* (3.A.-.-.-), which hydrolyze ATP to drive the active transport of solutes, includes just one protein [SwissProt: P33897]. *Recognized transporters of unknown biochemical mechanism* (9.A.-.-.-) comprises five proteins, of which three are members of the Transporter superfamily clan.

#### Enzymes

**Fig. 3:**
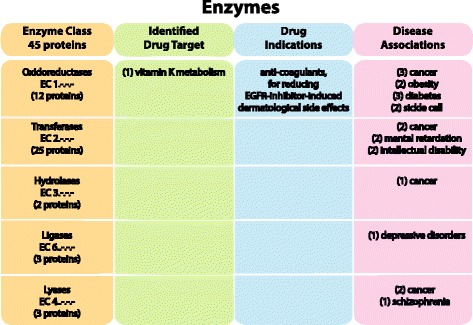
The human 4TM Enzyme class. The figure displays the 45 proteins identified with an EC number that belong to the Enzyme class. The enzymes are divided into five groups which correspond to the type of chemical reaction that they catalyze. Uniprot cross-references the ENZYME nomenclature database and IntEnz (Integrated relational Enzyme database) to obtain the EC number associated with a protein. The single protein classified as a drug target as well as the drug indications are displayed. The drug target and drug indications were identified through an updated dataset of all current targeted and potential proteins and genes involved in drug studies or experimentation. Some of the most common gene-disease associations are also shown for each of the subgroups in the Enzyme class. Three different resources were used to identify gene-disease associations: the Online Mendelian Inheritance in Man (OMIM) database; the Functional Disease Ontology (FunDO) resource; and the Jensen Lab Diseases database (see Methods for details). Proteins that are involved in enzymatic activity but do not have an associated EC number are not included in this class, but rather in a subgroup of the Miscellaneous proteins. The number in parenthesis represents the number of proteins that have been identified

The Enzyme class includes 45 proteins with a corresponding Enzyme Commission (EC) number (Fig. 3). *Oxidoreductases* (EC 1.-.-.-) that catalyze oxidation/reduction reactions in which H and O atoms or electrons are transferred from one substance to another include 12 enzymes. There are varied specific functions within this group, including iron ion binding and lipid metabolic processes [28]. *Transferases* (EC 2.-.-.-) are the largest enzymatic group with 25 proteins, and 18 of these contain the zf-DHHC palmitoyltransferase functional domain (PF01529) which is involved in zinc as well as other ion binding [31]. While there are 23 mammalian DHHC proteins identified in the membrane proteome [32], only 18 of them are predicted to have 4TMs. There are two proteins described as *hydrolases* (EC 3.-.-.-), which use hydrolysis to form two products. Three proteins are *lyases* (EC 4.-.-.-) and two of these contain the Protein tyrosine phosphatase-like protein domain (PF04387) which functions in very long chain fatty acid biosynthesis [31]. All three lyases are involved in a variety of disease conditions. And three proteins are classified as *ligases* (EC 6.-.-.-) in which all are involved as E3 ubiquitin ligases.

#### Dual functions

**Fig. 4:**
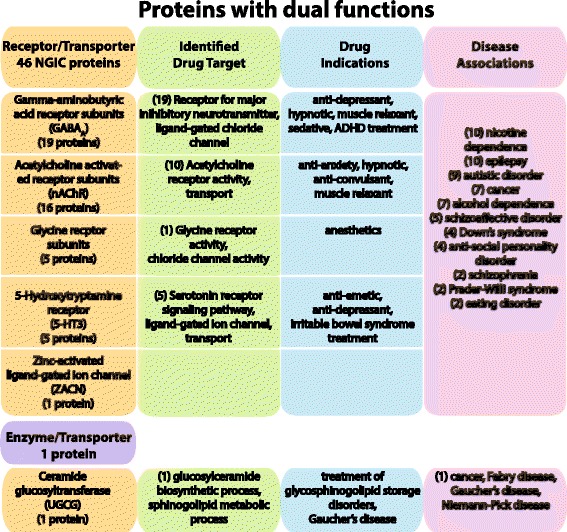
Proteins identified in the Dual functions class. The figure shows the 47 proteins identified as having specific dual functions. All of the neurotransmitter gated ion channel (NGIC) proteins are presented here, which include 46 members that are characterized by being a transporter and having a TC number (1.A.-.-.-; *alpha-type channel*) as well as being identified as a receptor. The NGIC are divided into their five different protein families, and the number of proteins identified as drug targets and the drug indications are presented. In addition, one protein is determined to be both an enzyme and transporter and the information is presented in the bottom row of the figure. The drug target and drug indications were identified through an updated dataset of all current targeted and potential proteins and genes involved in drug studies or experimentation. The gene-disease associations for all NGIC proteins are also displayed. Three different resources were used to identify gene-disease associations: the Online Mendelian Inheritance in Man (OMIM) database; the Functional Disease Ontology (FunDO) resource; and the Jensen Lab Diseases database (see Methods for details). The number in parenthesis represents the number of proteins that have been identified

Forty-seven proteins are distinguished as having dual functions as either receptor/transporter (46 proteins) or enzyme/transporter (1 protein) (Fig. 4). The complete repertoire of the 4TM neurotransmitter gated ion channel family, also known as the anionic and cationic cys-loop receptor group, includes all 45 proteins that belong to four different families as well as the single zinc activated ligand-gated ion channel protein [SwissProt: Q401N2]. The four families include gamma-aminobutryic-acid (GABA_A_), glycine, 5-hydroxytryptamine-3 (5HT3), and acetylcholine (nAChR) receptors and each protein contains the conserved neurotransmitter ligand binding domain (PF02931) as well as the ion channel domain (PF02932). All 46 NGIC proteins are described with a TC number as well as being identified as a receptor. In addition, these proteins are characterized by extracellular N- and C-termini. Thirty-five of the proteins are identified as drug targets which include a range of indications, as shown in Fig. 4. The common disease conditions include neurological diseases such as epilepsy, autistic disorder, schizophrenia, Alzheimer’s disease, various dependencies, as well as different cancers.

#### Receptors

The two proteins identified solely as receptors each belong to different receptor types. The first receptor is the high affinity immunoglobin epsilon receptor subunit beta [SwissProt: Q01362] and is a member of the IgE receptors, which combine with specific molecular sites on the surface of B- and T-lymphocytes. This protein is also identified as a drug target and is used as an anti-asthmatic agent. The second receptor is the type-1 angiotensin II receptor-associated protein [SwissProt: Q6RW13] and is a cell surface protein that binds angiotensins, which cause vasoconstriction that increases blood pressure, and activates intracellular changes that influence cell behavior. The Receptor class was identified using manual screening with two resources: the Medical Subject Headings (MeSH) [33] database and IUPHAR/BPS: Guide to Pharmacy [34].

#### Miscellaneous

**Fig. 5:**
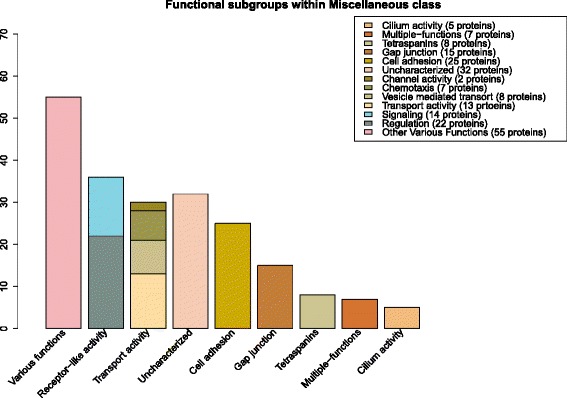
The functional subgroups of the Miscellaneous class. The graph displays the breakdown of the larger functional subgroups that contain 158 of the 213 proteins that belong to the Miscellaneous class, as well as the remaining 55 proteins under Various functions. The subgroups were determined by individually examining GO (general) and GO (molecular) terms as well as Pfam domain and protein family descriptions to categorize proteins with similar functions. Many proteins participate in multiple activities, and the subgroups are meant to highlight some of the main functional tasks within the dataset and how many proteins are involved with them. The Transport activity column includes the four subgroups *transport activity, vesicle mediated transport, chemotaxis*, and *channel activity* and totals 30 proteins altogether. The Receptor-like activity column includes both the *signaling* and *regulation* subgroups and totals 36 proteins as well

Many of the 213 proteins classified as Miscellaneous are multi-functional and involved in a variety of activities. Fourteen of the Miscellaneous proteins have signal peptides predicted and five proteins have extracellular terminal locations predicted. Fig. 5 shows several of the larger subgroups of proteins with similar functions that have been categorized using GO terms. The subgroups are meant to show the broad activities within the dataset and present some of the main functional tasks and how many proteins are involved in them. These functional subgroups can be composed of a single protein family, for example all 15 proteins associated with *gap junctions* are connexins, or be characterized by several different families, such as the 25 proteins involved in *cell adhesion* (in which 22 are claudins and 3 are tetraspanins).

Two of the Miscellaneous proteins are identified as drug targets. One is Gap junction alpha 1 protein [SwissProt: P17302], which is a connexin subunit and plays a possibly critical role in hearing and in bladder functional capacity [28]. It is being investigated for use as a wound healing accelerant [35]. The other identified drug target is Leukocyte antigen CD37 [SwissProt: P11049] – a tetraspanin that is involved in negative regulation of cell proliferation and positive regulation of immunoglobulin production, amongst other functions [28]. This protein is being targeted as a possible anti-neoplastic agent in cancer treatment.

#### Uncharacterized proteins

**Fig. 6:**
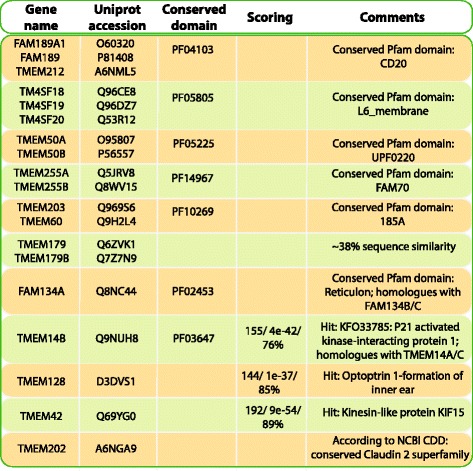
Uncharacterized proteins. This figure presents the six sets of uncharacterized proteins that have multiple members with the same conserved Pfam domain or high sequence similarity. In addition, five uncharacterized proteins that have either conserved domains or hits through BLASTP searches are also shown

Thirty-two of the Miscellaneous proteins have an uncharacterized function and twenty-four of these proteins have a recognized Pfam domain. Within the uncharacterized proteins, there are six interesting clusters of sequences: there are two sets that each have three members with the same conserved domain (PF04103 and PF05805); three sets of proteins with two members each that have the same domain (PF05255, PF14967, and PF10269); and a sixth group with two proteins that do not contain a conserved domain, but share ~38 % sequence similarity and belong to the same protein family. (See Fig. 6 for details). In addition, there are three proteins that have possible homologue hits through BLASTP searches and two proteins that have conserved domains that were identified through the Conserved domain database; the Reticulon domain and the Claudin 2 superfamily, which contains the claudins. Several of the clusters and homologue hits are discussed below, because while the uncharacterized proteins do not have any associated function ascribed to them through GO terms, valuable information was gleaned through literature searches.

TMEM50A and TMEM50B [SwissProt: O95807 and P56557] both contain the UPF0220 Pfam domain (PF05255) and are the sole members of the TMEM50 protein family. TMEM50A is located on chromosome 1p36.11 in the RH gene locus, between the RHD and RHCE genes, where its position may be linked to RH haplotypes and contribute to selective pressures regarding certain RH haplotypes [36, 37]. According to the Gene Expression Omnibus (GEO) [38], TMEM50A appears to be highly upregulated in late stage cervical cancer in comparison to normal cells. In a study that assesses biomarkers to investigate the etiology of Down syndrome, TMEM50B was found to be upregulated two fold in Down syndrome samples compared to normal [39].

TMEM14B [SwissProt: Q9NUH8] is a member of the TMEM14 protein family which includes TMEM14A, TMEM14B, TMEM14C, TMEM14D (which is a pseudogene), and TMEM14E, of which TMEM14B and TMEM14C are predicted as 4TMs. According to a recent 2015 article [40], NMR shows that 4TMs are predicted in TMEM14A and TMEM14C, but only 3 TMs span the entire membrane. Perhaps this contributes to TMEM14C as one of the few proteins with the N-terminus predicted to be located in the extracellular environment. TMEM14A/B and C are located on chromosome 6 and TMEM14E is located on chromosome 3. Only one gene, TMEM14C, has any associated function – heme biosynthetic process activity; all others have yet to be characterized. However, in yeast TMEM14A stabilizes the mitochondrial membrane potential and inhibits retinamide-induced apoptosis [41].

Neither TMEM179 nor TMEM179B [SwissProt: Q6ZVK1 and Q7Z7N9] contain a conserved Pfam domain but they share ~38 % sequence identity and belong to the same protein family. No function could be found associated with these proteins, however an article from 2009 [42] identifies TMEM179, which is located on chromosome 14q32.33, as an ‘evolutionary breakpoint’ region. This region is repeat rich and ‘reused’ during karyotypic evolution in that breakpoint features that are retained may have predisposed these genomic regions to large scale chromosomal instability.

TMEM203 and TMEM60 [SwissProt: Q969S6 and Q9H2L4] both contain the Pfam domain TMEM185A (PF10269), which is also conserved in the proteins TMEM185A and TMEM185B (neither of these are predicted as 4TM). This domain has been identified to be involved with the Fragile-X syndrome through the protein TMEM185A [43], however TMEM203 and TMEM60 share only ~10 % identity to the TMEM185A/B proteins and ~21 % identity between each other. In a recent publication, the previously uncharacterized TMEM203 protein was described as an evolutionarily conserved regulator of intracellular calcium levels that is required for spermatogenesis [44].

FAM189A1, FAM189B, and TMEM212 [SwissProt: O60320, P81408, and A6NML5] each contain the CD20 domain (PF04103), which is a member of the Tetraspanin-like clan. All three are uncharacterized, however FAM189B has been shown to possibly interact with the WW domain binding and be involved in protein binding [45]. Additionally, the three proteins TM4SF18, TM4SF19, and TM4SF20 [SwissProt: Q96CE8; Q96DZ7; Q53R12] all contain the L6_membrane domain (PF05805), which has unknown functions. However, there are three other proteins in the TM4SF protein family with the L6 domain and two are involved in regulation and signaling while the third is a transporter.

### Major Pfam families and clans within 4TM dataset

**Fig. 7:**
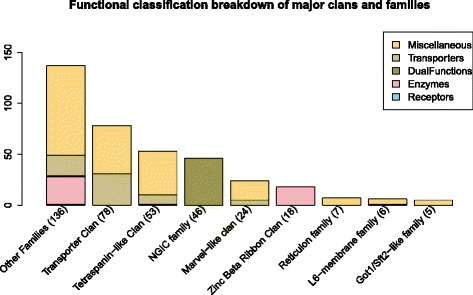
Functional classification breakdown within the 4TM major clans and families. The figure displays the breakdown of how many proteins within each major clan and family are identified for each functional class, i.e. Transporter, Enzyme, Dual function, Receptor, and Miscellaneous classes. Approximately 61 % of the dataset is described by these eight clans and families. The number in parenthesis corresponds to the total number of proteins within that clan or family

There are eight major Pfam domain families and clans within the dataset that contain between 5–81 members (see Fig. 7): Transport superfamily clan (78 proteins), Tetraspanin-like clan (53 proteins), Marvel-like clan (24 proteins), Zinc beta ribbon clan (18 proteins), NGIC family (46 proteins), Reticulon family (7 proteins), L6-membrane family (6 proteins), and Got1/Sft2-like family (5 proteins). The clans are composed of homologous domain families and include the unique four-transmembrane protein families such as claudins, connexins and tetraspanins. The domain families are formed from the collection of proteins with the same conserved Pfam domain, such as the 46 members of the NGIC family, and which do not belong to any clan and thus do not have any identified homologous sister families.

The largest clan, the Transport superfamily clan, comprises 78 proteins from six different conserved Pfam domain families. The six families within this clan include: the aforementioned connexin domain (PF00029, 21 proteins); PMP22_claudin (PF00822, 37 proteins); Claudin_2 (PF13903, 10 proteins); L_HGMIC_fpl (PF10242, 5 proteins); GSG-1 (PF07803, 2 proteins); and innexin (PF00876, 2 proteins). The predominant functions include cell adhesion, transporter activity, regulation, and cell communication via gap junctions.

The Tetraspanin-like clan is the second largest clan with 53 proteins and is composed of two families: the ‘true’ tetraspanin domain family (PF00335, 33 proteins) and the CD20 domain family (PF04103, 20 proteins). The ‘true’ tetraspanins are involved in transport activity and have also been identified as building tetraspanin enriched domains (TEMs) that facilitate protein scaffolding and assembly of specialized complexes [26]. The other domain family, CD20, appears to be cell-surface proteins that are involved in important cell regulation and differentiation activities, as well as possibly facilitate intracellular protein-protein interactions [46].

The 24 proteins that contain the MARVEL domain belong to the Marvel-like clan. Five of the MARVEL proteins are classified as transporters while others have shown association with specialized membrane microdomains (rafts) that could be involved in cholesterol-rich membrane apposition events in cellular processes such as biogenesis of vesicular transport carriers or tight junction regulation [47]. The eighteen proteins in the Zinc beta ribbon clan contain the zf-DHHC domain and act as transferases. The 46 members of the NGIC family has been discussed previously and the seven proteins in the Reticulon family are involved in activities such as transport and regulation as well as the apoptotic process, but one of them is also completely uncharacterized. The last two domain families include the six proteins of the L6-membrane family in which several are involved in transport and regulation, however three of them are also completely uncharacterized, and the five proteins of the Got/Sft2-like proteins that function in vesicle mediated transport.

Some members of these homologous families have been well-studied; however there are others that are still not fully described. For example, of the 27 members of the claudin family, only limited functional knowledge is available on at least 13 of the proteins: *Claudins 6, 9, 12, 13, 18, and 20–27* [48]. Additionally, meaningful GO functional terms are lacking for at least five proteins of the ‘true’ tetraspanins (*Tspan11, Tspan13, Tspan16, Tspan18, and Tspan19*), as well as at least eleven proteins that contain the CD20 domain and are members of the MS4A protein family which is involved in important cell regulation and differentiation activities.

### Disease-gene and cancer associations and drug targets

There are 215 genes identified with disease conditions. Various types of cancers, such as lung, colon, breast, and liver, are the most common diseases identified with 72 genes recognized in association with them. Many of the genes are described in multiple disease conditions, as can be expected from the critical functional activities that membrane proteins participate in. There are 14 genes associated with epilepsy, 13 genes with schizophrenia, and 13 with autism. Conditions involving deafness are associated with 12 genes; 10 genes are involved with nicotine dependence; diabetes, Alzheimer’s disease and alcohol dependence each have 7 genes identified with them. As NGIC receptors are involved in a plethora of neurological disease conditions, it is not surprising that disease conditions such as schizophrenia, autism, Alzheimer’s and dependence/addiction are heavily represented. Additional file 1 includes the complete gene-disease association descriptions. There are 44 proteins identified as a current targeted or potential protein that is involved in drug studies or experimentation, with 35 of them identified in the NGIC family.

### Topology

**Fig. 8:**
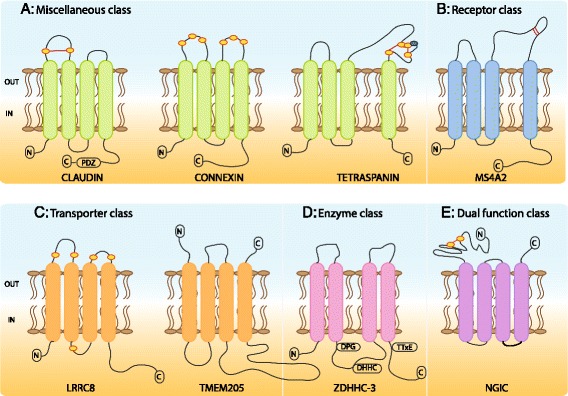
(Parts **a**, **b**, **c**, **d**, and **e**). Common topologies and conserved features within the 4TM dataset. Part **a** The Miscellaneous class includes proteins that have been characterized into subgroups through similar functional activities. Common features include intracellular termini and conserved cysteine residues (yellow outlined in red ovals) in the extracellular loops that either engage in forming disulphide bonds (e.g. claudins and tetraspanins) or interact and form bonds with other proteins (i.e. connexins). Tetraspanins have 4-6 conserved cysteine residues as well as a conserved CCG (cysteine-cysteine-glycine) motif in the large extracellular loop 2. Part **b** The example here, MS4A2, is one of the two identified receptors and a member of the MS4A protein family, in which 16 members are characterized by 4TMs, a CD20 domain, and an in N-terminus. Part **c** The Transporter class includes 66 proteins, of which 65 have an in N-terminus and conserved cysteine residues in the extracellular loops are common. TMEM205 is the sole transporter with the opposite topology, and is interesting due to its use of novel mechanisms in cisplatin (chemotherapy) resistance [82]. Part **d** Of the 45 Enzyme class proteins, all except five maintain an N-terminus intracellular location. ZDHHC-3 is a typical protein of the ZDHHC protein family, characterized by 4TMs, a conserved DHHC domain, and a conserved DPG (aspartate-proline-glycine) motif as well as a TTxE (threonine-threonine-asparagine-glutamate) motif. Part **e** The Dual function class contains 47 proteins and all 46 proteins of the neurotransmitter gated ion channel family (NGIC) are included here. The NGIC family has a long extracellular N-terminus that contains several important binding sites as well as two conserved cysteine residues that participate in disulphide bonds. The NGIC family is unique in that it has extracellular N- and C-termini and also has signal peptides predicted in 40 of the proteins

In addition to the number of membrane spanning helices, the orientations of the N- and C-termini are important factors in determining the functional activity of the protein. The terminal orientations are usually determined by the initial insertion of the peptide into the membrane, however the presence of a signal peptide can influence different orientations. Signal peptides are short sequences of amino acid residues attached to the N-terminus domain that target the protein to the membrane and are then subsequently removed by proteolysis post membrane insertion. In addition, when using transmembrane protein prediction methods, it is important to assess and excise signal peptides from sequences as otherwise they can be mistaken as transmembrane helices due to the hydrophobicity of the peptide sequence [49]. The results of the TOPCONS-single topology predictions for this dataset include 316 proteins with the N- and C-termini located within the lumen of the membrane and 57 proteins with the terminals in the outside environment. The complete NGIC group, which compose virtually all of the Dual function class with 46 proteins, have the N- and C-termini located in the extracellular environment. The extracellular N-terminus is consistent with the other large receptor groups, i.e., the 1TMs and 7TMs, however the C-terminal is usually located in the intracellular environment due to the important activities it is typically involved in, particularly signaling transduction. Additionally, 40 of the NGIC proteins are predicted from the SignalP signal peptide prediction software to have signal peptides. In total there are 60 proteins that are predicted to have signal peptides. As current transmembrane protein prediction methods are based on classical helical structures, i.e. those that completely span the membrane, they do not take into account anomalies such as reentrant loops, short breaks in helices, and helices that lie along the surface of the membrane [8]. As mentioned previously with TMEM14C, this method limitation could possibly affect the topology prediction. For example, those 11 proteins that have predicted extracellular orientation (excluding the NGIC family) might be interesting proteins to study for transmembrane structural purposes. Examples of common structures and topologies for each functional class are represented in Fig. 8, which highlights conserved features and N- and C-terminus orientations found in the 4TM dataset. For example, claudins, tetraspanins, and connexins categorized in the Miscellaneous class are shown with the four conserved membrane regions, the intracellular location of the N- and C-termini, and also conserved cysteine residues that are often found in the extracellular loops and are involved in forming disulphide bonds.

## Discussion

This consensus topology based screening of 4TMs followed by manual curation presents the most robust and updated dataset of the 4TM complement of the human proteome. Our initial screening of the human proteome predicts 555 sequences with four membrane-spanning helices and this estimate is comparable with the human tissue-based Protein Atlas database that currently predicts 554 sequences with four-transmembrane regions [50]. Both of these 4TM prediction datasets include all protein products (such as isoforms), and the Protein Atlas database also uses a majority decision method but with seven different prediction methods based on the Ensembl gene annotation. Our protein dataset consists of 373 members after manual curation and also appending 40 selected proteins, which represents 7 % of the ~5550 human membrane proteome. This dataset contains 69 more predicted proteins that what was previously estimated in our human membrane proteome, which also accounted for a single protein product from each gene, obtained from the IPI human version 3.39 in 2009. The different number of predicted proteins might be attributed to our manual curation process, as well as different number and types of prediction methods and different gene annotation sources. There are 341 proteins characterized in the dataset, which amounts to approximately 91 % of the proteins having an associated description.

Transporters make up the largest category of 4TMs and consist of 37 % of the dataset, with 17 % identified solely in the Transporter class, an additional 12 % in the Dual function category, and 8 % more in the Miscellaneous class that are members of subgroups such as *transport activity, vesicle mediated transport, channel activity, and chemotaxis* (see Fig. 5). In the entire human membrane proteome [5], transporters compose approximately 15 % of the proteins, which shows that the 4TM dataset contains more than double that amount proportionally. And when comparing against the 6TM protein set, another membrane group known to be involved in transport activity, roughly 30 % of that group of proteins function in transport. Comparatively, 23 % in total of 4TM proteins are involved in receptor activity and 13 % function in enzymatic activity, which is commensurate to the roughly 25 % of receptors and 10 % of enzymes found in the entire membrane proteome. This might suggest that the four-transmembrane topological architecture is more favored for transporting molecules than over other functions.

Intriguingly, in our topology analysis we found an important exception to the ubiquitous membrane proteome intracellular locale of the C-terminus in the NGIC group that performs dual functions; all 46 members have extracellular N- and C-termini. And concomitantly, 40 of the 46 NGIC proteins are predicted to have signal peptides. It has been shown [8] that the prokaryotic 2TM glutamate receptor is an example of where the addition of a cleavable signal peptide can influence the terminal orientations of proteins that originally had a cytoplasmic N-terminus, particularly with proteins with a fewer number of transmembrane helices. The addition of a signal peptide can cause reorientation of the termini ends through causing the translocation of the N-terminus to the outside environment. When the signal peptide is subsequently cleaved, the location of the terminal is in the extracellular region [51]. Additionally, the NGIC family possesses a long (~200 amino acids) N-terminus tail which contributes to a stably folded ligand binding domain [52, 53]. As shown in the 7TM GPCR families, signal peptides are more common in those proteins that contain those two factors, i.e. a long N tail and an N-terminus that engages in stabilizing the ligand binding domain [54, 55]. This set of factors regarding the signal peptides and terminal orientations are interesting components that could possibly contribute to the evolution of the NGIC topology orientations.

An interesting aspect of the functional classification of the 4TM dataset is that 61 % of the proteins can be described by 8 large Pfam families or clans, containing a range from 5 to 81 members. Fig. 7 depicts the eight clans and domain families and displays the number of proteins within each functional class for each clan or family. Furthermore, there are only 12 proteins that do not contain any type of conserved functional domain, so up to 97 % of the 4TM dataset contains conserved features that are found in homologous domains within other proteins. As described in the results, some members of these homologous families have been well-studied, however many others have still not been investigated and function is inferred through homology. Additionally, this evaluation elucidates six groups (14 proteins) that have uncharacterized functional activity and that share not only conserved sequence features but also a similar 4TM structure (see Fig. 6). These groups of proteins indicate interesting avenues of future investigation.

Overall, we estimate that 58 % of the genes in our 4TM dataset are identified as being involved in various disease conditions and roughly 19 % of the genes are possibly involved in different types of cancer. It is interesting to note that proportionally the Transporter class has a higher percentage of genes that are possibly involved in cancer, with 29 % of the proteins in that class identified. In comparison, the Miscellaneous and Enzyme classes both had 17 % of their proteins identified with cancer. Part of this might be accounted for by more research on proteins involved in transport activities. The involvement of 4TM proteins in different types of cancer and other disease conditions point toward the importance of this less explored class of transmembrane proteins and it may be possible to further utilize this class of transmembrane proteins as drug targets.

## Conclusions

In conclusion, we have functionally classified and manually curated the 4TM complement of the human proteome, which is characterized by four-transmembrane helices and the majority of proteins containing conserved N- and C-termini intracellular location. This examination of the 4TM structural group and related functions shows the ubiquitous transport activity that is unique in a membrane group with such few transmembrane helices. While 4TM proteins are not necessarily identified as classical receptors and transporters, we show they are still heavily involved in these activities as well as cell communication, cell adhesion, and working as scaffolding and structural elements. This has led to perhaps an oversight in pharmacological research efforts. As this detailed characterization of the 4TM dataset and the associated gene-disease information exhibit, these proteins participate in a host of important activities and it is becoming more apparent that they are also involved in various disease conditions including cancer and neurological conditions. Further, this dataset highlights particular 4TM proteins that are waiting to be studied in connection to diseases and as possible drug targets.

## Methods

### Automatic prediction

#### Generating the initial 4TM dataset

A two-step analysis process was used: *in silico* transmembrane prediction (Fig. 1a) followed by manual curation and classification. The *Homo sapiens* genome assembly GRCh38 with genome annotation GenCode v21 translations was downloaded from GenCode [56] on February 10, 2015. The genome annotation file contained 93,139 gene products from 19,881 protein-coding genes. As transmembrane protein prediction methods have difficulty differentiating between N-terminal alpha helices and cleavable signal peptides, the genome annotation file was evaluated with a standalone version of SignalP 4.1 software [49]. SignalP uses a neural network based method to distinguish between N-terminal helices and signal peptides. The parameters used were: eukaryotic organism, default minimum signal peptide length of 10aa, the best method was chosen which designated TM regions might be present, and the default cutoff value of 0.45. The mature sequences with the signal peptides excised were then evaluated with TOPCONS-single transmembrane prediction web server to assess membrane topology including the number of membrane-spanning helices and orientation of the terminal ends [57]. As prediction methods use different algorithms to discriminate transmembrane helices, the number of alpha helices predicted by each method varies. One manner to improve the accuracy in membrane prediction and is to use several prediction methods and make a consensus or majority decision regarding topology. TOPCONS-single uses a consensus decision method that comprises five different prediction tools: SCAMPI-single [58]; S-TMHMM [59]; MEMSAT 1.0 [60]; HMMTOP [61]; and Phobius [62]. The *Benchmark of membrane helix predictions from sequence* website was used to assess the accuracy of the methods used in TOPCONS-single, and the percentage accuracy for which all membrane helices were correctly predicted for the methods ranged from 56–72 % [63], which is comparable to the TOPCONS-single published benchmark results (51–73 %) [57]. The initial membrane topology was performed using all five prediction methods. The predicted four-transmembrane sequences were retrieved and sorted by gene identification (Ensembl gene identification) and sequence length. The dataset was reduced by retaining the longest sequence per gene from the predicted sequences. At this juncture, the dataset possibly includes isoforms of proteins as the predicted 4TM sequence may not necessarily be the canonical sequence of that gene. The dataset was then re-evaluated with TOPCONS-single again to attempt to mitigate false-positive proteins and obtain as accurate dataset as possible, and the final non-redundant predicted 4TM dataset was produced.

### Manual curation

#### Determining the unambiguous 4TM proteins

The manual classification process included four steps, which are highlighted in Fig. 1B. Universal protein resource (Uniprot) [28] was used to access annotation information on the dataset by using the unique Ensembl protein identification associated with each protein. Uniprot is a large comprehensive repository of protein sequences with manually curated as well as automatically generated associated annotations. For each protein, the associated UniProt annotations were used in the curation process: *gene name, sequence status, review status, Consensus Coding Sequence identifier, Transporter Classification number, Enzyme Commission number, Pfam domain information, Gene Ontology annotation terms, and protein family information*.

The Uniprot sequence information is derived from translated sequences that have been submitted to the International Nucleotide Sequence Database Collaboration (INSDC), which is EMBL-bank, GenBank, and DDBJ. The canonical sequence is determined from either the most prevalent, the most similar to orthologous sequences, the properties of the amino acid composition, or in lieu of nothing else, then the longest sequence. The *sequence status* is defined as either complete or fragment, in which the canonical sequence is missing amino acid residues, often in the initial or terminal ends. Those protein sequences identified as fragments were considered invalid proteins and culled from the dataset. To reduce false-positive predictions from TOPCONS-single, proteins that were identified in literature or database sources as having less than or greater than four-transmembrane segments were also removed from the dataset.

The initial gene annotation source, GenCode, has ~1050 more protein-coding gene entries than the most conservative annotation resource, the Consensus Coding Sequence dataset (CCDS) [64]. Therefore the CCDS identifier was used to assess the validity of each protein, i.e. that each has acceptable transcriptional support and recognized protein-coding annotation. Additionally, the sequence status, CCDS identifier, and sequence length were used to ensure that the predicted protein was the main (or canonical) protein product of the gene and not an isoform. There are eight predicted proteins in the dataset that do not have a CCDS identifier associated with them. Five of these have annotation support through RefSeq [65], Vega [66], UCSC, and Ensembl [67] and the authors have chosen to retain them in the dataset. Two genes are identified as pseudogenes (*Gje1* and *Cyp2d7*) however they appear to be capable of some functional activity and were retained in the dataset: while *Gje1* does not form functional gap junction channels, it causes enhanced ATP release from HeLa cells, and with two SNPs *Cyp2d7* may result in an open reading frame with a protein-coding product [65]. *Unc93b1* was discussed in our previous examination of the membrane proteome [5] and has been evaluated in literature as having multiple functions [68] with truncated isoforms and thus could be an important protein to include.

The functional domains and families for each sequence were obtained through cross-referencing the Pfam database. The Pfam [69], InterPro [31], and ProSite [70] databases as well as appropriate literature were used to assess the characteristics and number of transmembrane helices of each Pfam domain associated with each protein. Proteins that contained a domain that had evidence of more than four-transmembrane helices, and thus the predicted 4TM protein contained an incomplete functional domain, were also discarded from the dataset. The remaining proteins compose the 4TM unambiguous dataset.

#### Classifying the unambiguous 4TM proteins

The Transporter Classification number (TC), Enzyme Commission number (EC), Gene Ontology (GO) terms, Pfam domain characteristics, and protein family information were used to describe the functions of the unambiguous 4TM dataset and categorize them into appropriate classes. The Transporter Classification Database [27] is cross-referenced by Uniprot to provide all TC numbers. The Transporter Classification system is an IUBMB approved classification system for membrane transport proteins that includes both functional and phylogenetic information. Proteins that had an associated TC number were classified as Transporters. The Enzyme Commission number is produced by cross-referencing the ENZYME nomenclature database [71] and IntEnz (Integrated relational Enzyme database [72]. If a protein had an associated EC number, they were categorized as Enzymes. Receptors were determined though manually processing selected proteins through the Medical Subject Headings (MeSH) [33] and IUPHAR/BPS: Guide to Pharmacy [34] resources.

To help mitigate possible false-negative hits from TOPCONS-single that missed true 4TM proteins and to provide as complete a 4TM protein repertoire as possible, missing proteins from known protein families (such as claudins and tetraspanins) were manually explored and added to ensure the families were fully represented in the dataset. In total, forty additional proteins were added to the final dataset that had been identified in literature or database resources as containing 4TM regions.

Proteins that were not classified as Enzymes, Transporters, Receptors, or Dual Functions were grouped into the Miscellaneous class. To further describe the miscellaneous proteins, GO (general) and GO (molecular) terms were used to categorize proteins with similar functions into subgroups such as *cell adhesion, chemotaxis, gap junction, enzymatic activity, regulation*, etc. The GO terms are cross-referenced from the GO project, which aims for a consistent and comprehensive functional annotation for gene products across databases [73]. There were several proteins that did not have any associated GO terms, but there were Pfam domains that described the function of the protein. There were also proteins where even with a conserved Pfam domain the function of the protein was unknown or uncharacterized. Those miscellaneous proteins that did not have any described function were categorized as uncharacterized.

#### Investigating the uncharacterized proteins

The uncharacterized proteins were further researched using the National Center for Biotechnology Information (NCBI) BLASTP [74] resource. BLASTP was used to determine if there were any possible homologues with the uncharacterized protein. The Non-redundant protein dataset (nr) was the search dataset and the default parameters were used including the Blosum 62 substitution matrix; Word size: 3; and Expectation threshold: 10. Resulting hits with acceptable scores for investigating distant homologues were retained: greater than ~25 % sequence similarity; an E-value between 0 –1e-6; and a bit score value >50 [75]. In addition, literature searches through NCBI PubMed were performed on selected proteins.

#### Determining gene-disease associations and identifying drug targets

Three different resources were utilized to investigate gene-disease associations within the dataset. The Online Mendelian Inheritance in Man (OMIM) [76] database was cross-referenced through UniProt and the associated annotations for each protein were downloaded. OMIM contains a catalogue of genetic traits and disorders with referenced overviews on all known Mendelian disorders. The Functional Disease Ontology (FunDO) [77] resource incorporates Disease Ontology terms, which identifies gene-disease associations, but has a simplified vocabulary list called DOLite to enable more interpretable results [78]. The DOLite gene-disease mapping file was downloaded and gene names within the dataset were searched. The third resource was the Diseases database [79] which incorporates disease-gene associations from automatic text mining, manually curated literature, cancer mutation data, and genome-wide association studies with evidence confidence scores for each association [80]. The filtered (non-redundant) text mining file was downloaded and searched to identify gene-disease associations within the dataset.

An updated dataset of all current targeted as well as potential proteins and genes involved in drug studies or experimentation was obtained from Rask-Anderson et al. [35]. This dataset is based on data from DrugBank [81], which provides extensive drug data and target information, and then has been manually curated to create a comprehensive non-redundant dataset. Both the proteins and genes within the unambiguous 4TM dataset were investigated to identify drug targets.

## Additional file

Additional file 1: Contains the UniProt identifications, gene symbols, and Ensembl protein identifications for the classification of the 494 valid 4TM dataset. Also included within the table is the following information: signal peptides, topology, Pfam domains, functional classifications, review status, CCDS identifier, and gene-disease associations. (XLSX 176 kb)

## Abbreviations

4TM: four-transmembrane helix containing protein
NGIC: neurotransmitter gated ion channel
TC: Transporter classification number
EC: Enzyme commission number
GO: Gene ontology
